# Bacterial sexually transmitted infections in France: recent trends and patients’ characteristics in 2016

**DOI:** 10.2807/1560-7917.ES.2019.24.5.1800038

**Published:** 2019-01-31

**Authors:** Ndeindo Ndeikoundam Ngangro, Delphine Viriot, Nelly Fournet, Corinne Pioche, Bertille De Barbeyrac, Agathe Goubard, Nicolas Dupin, Béatrice Berçot, Sébastien Fouéré, Isabelle Alcaraz, Michel Ohayon, Nathalie Spenatto, Chantal Vernay-Vaisse, referents for the regional offices of the French national public health agency, Josiane Pillonel, Florence Lot

**Affiliations:** 1 Santé publique France (the French national public health agency), Saint-Maurice, France; 2 National Reference Centre for bacterial STI (Chlamydia), *Pellegrin Hospital*, Bordeaux, France; 3 National Reference Centre for bacterial STI (Gonorrhoea), Alfred Fournier Institute, Paris, France; 4 National Reference Centre for bacterial STI (Syphilis), Cochin Hospital, Paris, France; 5 National Reference Centre for bacterial STI (Gonorrhoea), Saint-Louis Hospital, Paris, France; 6 Saint-Louis Hospital, Paris, France; 7 Dron Hospital, Tourcoing, France; 8 “Le 190”, Sexual Health Centre, Paris, France; 9 University Hospital Centre, Toulouse, France; 10 Departmental committee of Bouches-du-Rhône, Marseille, France; 11 Members of Cire referents group are listed at the end of this article

**Keywords:** sexually transmitted infections, Chlamydia, syphilis, gonorrhoea, MSM

## Abstract

Diagnoses of bacterial sexually transmitted infections (STI) have been increasing in France since their resurgence in the late 1990s. This article presents recent epidemiological trends until 2016 and the patients’ characteristics. STI surveillance relies on sentinel networks: a clinician-based network RésIST (clinical, biological and behavioural data for early syphilis and gonorrhoea), the lymphogranuloma venereum (LGV) network (clinical, biological and behavioural data for rectal LGV, and the laboratory networks Rénachla and Rénago (demographic and biological data for chlamydial infections and gonorrhoea, respectively). Here we describe trends between 2014 and 2016, using data from diagnostic centres which participated regularly during the study period. The number of early syphilis, gonorrhoea and LGV diagnoses increased between 2014 and 2016, particularly in men who have sex with men. An increase in syphilis and gonorrhoea cases was also observed in heterosexuals. Nevertheless, we observed a drop in 2016 for syphilis and chlamydial infections after two decades of increases. Under-reporting and shortage of benzathine penicillin in 2016 may explain this latest evolution. Regular screening of patients and partners, followed by prompt treatment, remains essential to interrupt STI transmission in a context where human immunodeficiency virus (HIV) prevention has expanded towards biomedical prophylaxis.

## Introduction

Diagnoses of bacterial sexually transmitted infections (STI) have been increasing in France since the upsurge of gonorrhoea in 1998, the resurgence of syphilis in 2000 and the re-emergence of lymphogranuloma venereum (LGV) in 2003 [1]. A specific laboratory survey implemented in 2012 estimated the annual incidence of *Chlamydia trachomatis* and *Neisseria gonorrhoeae* infections at ca 77,000 cases (257/100,000 people aged 15–49 years) and 15,000 cases (39/100,000 people aged 15–59 years), respectively [2].

Because of their frequency, transmissibility, associated complications and their role in the transmission of human immunodeficiency virus (HIV), STI surveillance is vital to establish the course and evaluation of preventative actions. The French national screening recommendations foresee the following: systematic chlamydia testing for asymptomatic young women (< 25 years) and men (< 30 years) visiting STI clinics or family planning centres from 2003 to 2008 and any health facility since a revision in September 2018; LGV testing for symptomatic patients or asymptomatic HIV-positive men who have sex with men (MSM) with a rectal chlamydial infection; opportunistic syphilis testing in exposed MSM, sex workers, HIV or LGV patients, migrants from endemic countries and people with multiple sex partners; and targeted gonorrhoea testing for MSM, HIV and/or STI patients who had unprotected sex [3-7]. Moreover, recent evolutions in HIV prevention with the 2016 launch of pre-exposure prophylaxis (PrEP) consisting of prophylactic antiretroviral prescription, and regular STI screening, might impact STI epidemiology, increasing the number of STI diagnoses [8].

In France, STI diagnostics is available at STI clinics (CeGIDD in French) for free, and paid by the national health insurance (which covers 95% of the population) at any level of the health system (private clinics, public hospitals or general practitioners (GP)). STI clinics, even if accessible by anyone, are designed primarily for high-risk and socially disadvantaged populations. After visiting a doctor in any health setting, patients with a prescription for diagnostic tests can choose to be tested in a private or public laboratory. Commonly, STI clinics do not charge a fee for biological tests, in contrast to other facilities where patients have to pay before cost reimbursement by the national health insurance.

The aim of this article was to describe epidemiological trends of bacterial STI in France from 2014 to 2016, and patients’ characteristics.

## Methods

**Figure 1:**
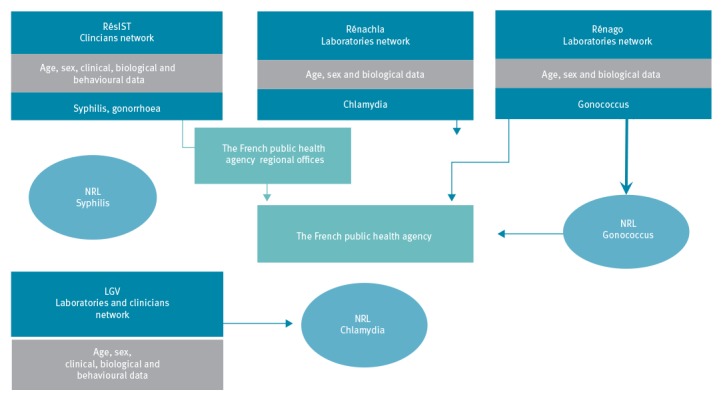
Organisation of the surveillance of sexually transmitted bacterial infections in France

LGV: lymphogranuloma venereum; NRL: national reference laboratory.

Because of insufficient exhaustiveness, the mandatory reporting of gonorrhoea, syphilis and LGV cases was replaced in 2000 by sentinel surveillance networks based on biologically confirmed case definitions (Figure 1) [1].

RésIST, the network of clinicians coordinated by Santé publique France (the French national public health agency), contributes to the surveillance of early syphilis (primary, secondary or early latent stages) and gonorrhoea. The collection of demographic, clinical, biological and behavioural data relies on practitioners working in different diagnostic centres (STI clinics, dermatological hospital consultation units, infectious illness consultation units, internal medicine or private medical practices). Nevertheless, the majority of syphilis (82.8%, n = 1,447) and gonorrhoea (95%, n = 2,389) cases reported in 2016 by the RésIST network were diagnosed in STI clinics and the coverage of this network is unknown. Standardised hardcopy forms are completed by physicians for each confirmed case.

Rectal LGV (infection due to the serovar L strain of *Chlamydia trachomatis*) is monitored via a sentinel network of laboratories, coordinated by the national reference centre (NRC) for chlamydial infections which collects biological and demographic data. They are completed with clinical and behavioural data collected through a standardised hardcopy form sent to the prescribing clinicians by the NRC.

Two laboratory-based sentinel networks, Rénachla and Rénago, gather demographic and biological data of cases with, respectively, chlamydial and gonococcal infection. The rates of coverage of these two networks were estimated at 18 and 23%, respectively, of the incident diagnoses confirmed in France in 2012 [2]. Since 2014, biologists have to fill a standardised online form instead of a paper questionnaire. Around half of the gonorrhoea patients in 2016 reported through Rénago (46.8%, n = 2,231) visited a GP, 35.3% (n = 1,683) a free STI clinic, 15.5% (n = 740) a hospital setting and 2.4% (n = 116) visited unknown diagnosis centres. Chlamydial infections reported through the Rénachla network were clinically diagnosed in STI clinics (25.6%, n = 3,369), by a GP (13.8%, n = 1,810), by other non-hospital specialists (9.5%, n = 1,257), in hospital settings (10.8%, n = 1,431) or in unknown diagnosis centres (40.2%, n = 5,287). The NRC for gonorrhoea plays a major role in the surveillance of resistance. It performs antimicrobial resistance tests (e.g E-tests, genotyping) on strains identified by 64 laboratories selected through the Rénago network.

In order to take fluctuations in network participation into account, recent trends between 2014 and 2016 are described here by taking into consideration only those diagnostic centres that consistently declared cases during these 3 years (79/119 Rénago laboratories; 35/49 Rénachla laboratories; 11/89 LGV centres; 78/125 RésIST syphilis centres; 59/112 RésIST gonorrhoea centres). Infection in several anatomical locations in the same individual counted as one infection. Patients’ characteristics (sex, age, residence, sexual orientation, presence of symptoms, type of specimen, HIV status, type of diagnostic centre) described, when information was available, here apply to the last year of the study period (2016). We chose the age groups in our analyses so as to match the national chlamydia screening recommendation (systematic screening for women < 25 years and men < 30 years).

LGV, RésIST and Rénago/Rénachla networks were approved by the French Personal Data Protection Authority (CNIL authorisation, respectively no. 10362, 902057 and 999298). Patients’ informed consent was required before any data collection. The number of people that opt out from this surveillance system by not providing their consent is unknown.

## Results

### Chlamydial infections

**Figure 2:**
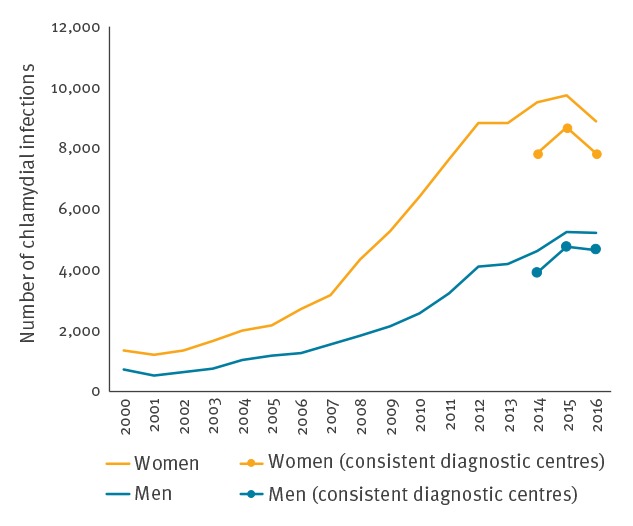
Number of chlamydial infections by sex, Rénachla network, France, 2000–2016 (n = 125,902)

The number of chlamydial infections increased by 15% from 11,733 to 13,438 diagnoses between 2014 and 2015 and declined by 7% from 13,438 to 12,495 diagnoses between 2015 and 2016. The overall increase in the period from 2014 to 2016 was more substantial in men (19% from 3,900 to 4,660 vs 0.03% from 7,833 to 7,835 in women) (Figure 2) and occurred in the Paris area (4% from 2,322 to 2,424) as well as in other metropolitan regions (7%, from 9,431 to 10,099) between 2014 and 2016.

In 2016, the majority of the 14,100 patients diagnosed with a chlamydial infection were women (63%, n = 8,890). The most affected age groups in women and men were, respectively, 15–24 year-olds (61%, 4,351/7,176) and 15–29-year-olds (67%, 2,521/3,774) (age groups here as per screening recommendations). Among the cases for whom symptom information was available (32%, 4,506/14,100), the percentage of diagnoses in asymptomatic patients decreased slightly from 59% (4,515/7,618) in 2014 to 45% (2,041/4,506) in 2016. The proportion of asymptomatic patients varied according to the place of consultation, from 26% (143/548) in hospital gynaecology units to 79% (1,105/1,399) in STI clinics. The main sample types which led to diagnoses were urine for men (79%, 4,091/5,210) and cervical-vaginal swabs for women (71%, 6,304/8,890); these latter percentages remained stable over the 3 years.

### Rectal lymphogranuloma venereum

**Figure 3:**
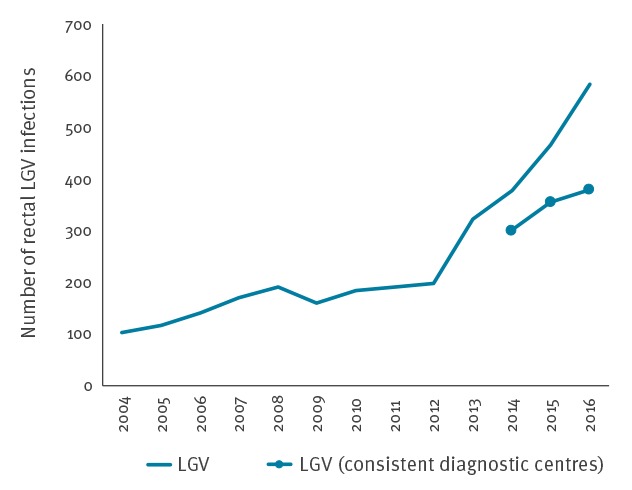
Number of rectal lymphogranuloma venereum, LGV Network – National centre for Chlamydia, France, 2002–2016 (n = 3,225)

Between 2014 and 2016, the number of rectal LGV cases increased by 26% from 301 to 380 (Figure 3). This STI affects almost exclusively MSM, who represented 95% (555/584) of the LGV cases reported in 2016. The most affected age group were 30–49-year-olds (58%, n = 344). The level of HIV co-infection remained high: 71% (346/484) of reported LGV-positive patients were HIV-infected when information was available. Most patients resided in the Paris area (61%, 356/584).

LGV: lymphogranuloma venereum.

### Gonorrhoea

**Figure 4:**
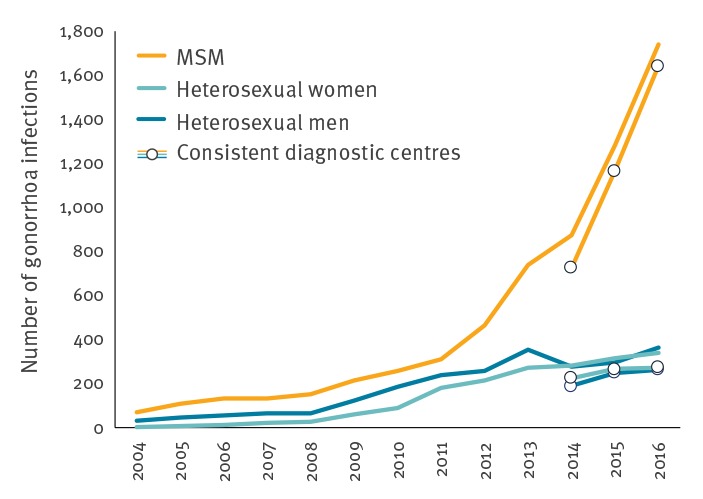
Number of gonorrhoea cases by sex and sexual orientation, RésIST network, France, 2004–2016 (n = 10,565)

Between 2014 and 2016, the number of gonorrhoea cases increased by 127%, from 724 to 1,641 diagnoses in MSM, by 21% from 224 to 270 diagnoses in heterosexual women and by 40% from 187 to 262 diagnoses in heterosexual men (Figure 4). This increase was observed in the Paris area and in other metropolitan regions. Of all gonorrhoea infections reported in 2016, 71% (1,740/2,434) concerned MSM. The most affected age groups in women and men were, 20–29-year-olds (respectively 58% (219/377) and 49% (1,053/2,140)), median age was 29 years (interquartile range (IQR): 21–38) in MSM, 25 years (IQR: 22–31) in heterosexual men and 22 years (IQR: 20–25) in heterosexual women. In 2016, the proportion of HIV co-infections remained stable at ca 14% (337/2,389); the highest proportion was observed in MSM (19%, n = 315/1,691 with available information).

MSM: men who have sex with men.

Between 2014 and 2016, a very high proportion of isolates were still resistant to tetracycline (56% (624/1,115) in 2014 and 64% (665/1,040) in 2016) or to ciprofloxacin (43% (476/1,115) in 2014 and 41% (422/1,040) in 2016). The number of strains resistant to azithromycin declined from 69 in 2014 to 59 in 2016 and only one strain with high resistance to azithromycin was isolated in 2014. The number of isolates resistant to cefixime decreased between 2014 (11 isolates) and 2016 (six isolates). No strain resistant to ceftriaxone was isolated in France between 2014 and 2016.

### Early syphilis (less than 1 year duration)

**Figure 5:**
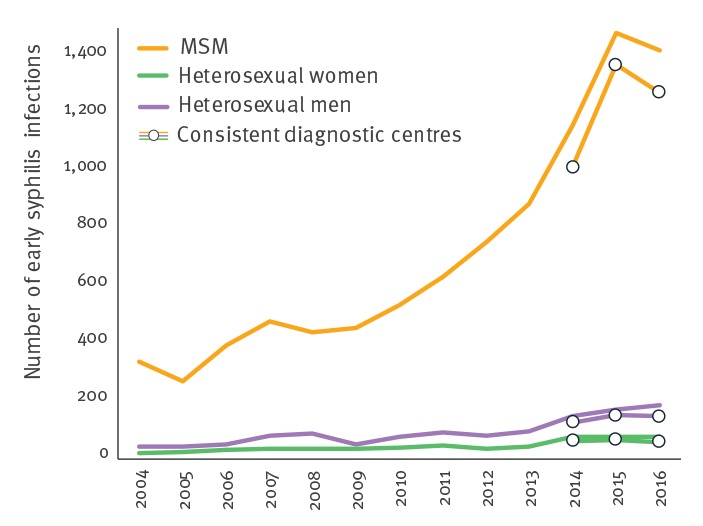
Number of early syphilis cases by sex and sexual orientation, RésISTnetwork, France, 2000–2016 (n = 12,112)

Between 2014 and 2015, the number of reported early syphilis cases in MSM rose by 35% from 1,015 to 1,371 diagnoses and then decreased by 7% to 1,274 diagnoses between 2015 and 2016 (Figure 5). This 2016 decrease point was observed after two decades of increases in metropolitan regions, irrespective of patients’ sexual orientation despite small case numbers in heterosexuals (217 in 2015 and 206 in 2016) [1,2].

MSM: men who have sex with men.

The distribution of early syphilis cases by stage (primary syphilis 27%, 468/1,749; secondary syphilis 31%, 539/1,749; and early latent syphilis 42%, 742/1,749) remained stable over the three years. Among patients with early syphilis in 2016, 81% (1,420/1,749) were MSM and only 5% (85/1,749) were women. Men aged 20–49 years were the most affected (79%, 1,307/1,655), irrespective of their sexual orientation, while most affected women were younger than 29 years (36/84 for whom information on age was available).

The level of HIV co-infection remained very high in syphilis patients in 2016, with approximately one third co-infected (33%, 551/1,648). The majority of co-infected patients were already informed of their HIV infection (92%, 505/551). Only 46 patients were diagnosed with HIV concurrently with their syphilis diagnosis. Co-infection was more common in MSM (36%, 484/1,358) compared with 23 heterosexual men and five women co-infected in 2016.

## Discussion

The number of early syphilis, gonorrhoea and LGV cases reported by the sentinel networks increased between 2014 and 2016. This trend was particularly noticeable in MSM in a context of increasing STI since the late 1990s [1,2]. A raise in syphilis and gonorrhoea diagnoses was also observed in the heterosexual population, although the number of cases was smaller.

A fifth of chlamydia and gonorrhoea cases (18% of chlamydial infections and 23% of gonorrhoea cases in 2012) are reported through the laboratory networks, assuming a steady coverage of sentinel networks since 2012; the reported patients are probably not representative of the general population [2]. This is mainly due to the voluntary participation of centres and the overrepresentation of STI clinics in the in the clinicians RésIST network. Moreover, no information is available to describe the patients who decline participation in a study, and the coverage of the clinician network RésIST is unknown.

The existing sentinel surveillance system reliably monitors epidemiological trends at a national level thanks to its consistently participating diagnostic centres. It shows an overall increase in the number of STI cases over two decades [1,2]. Nevertheless, compared with 2015, the number of early syphilis and chlamydial infections seemed to decrease in 2016. This trend change from 2015 to 2016 should be interpreted with caution on account of under-reporting, delayed reporting and no possibility to assess whether this decrease is statistically significant. Delayed reporting as an issue impacting on the number of cases was confirmed by a substantial number of questionnaires from 2015 and 2016 that we received after the end of study period. Several laboratories with a high level of activity reported difficulties to timely use the online individual questionnaire to report chlamydial infections. To maintain their participation to the network, they were invited to provide an electronic database, when it was possible, despite the loss of information for example about the presence of symptoms. A database uploading system might partly fix these problems and help sustain a good level of participation to the sentinel surveillance networks. Concerning the decrease in syphilis cases, the national shortage of benzathine penicillin in 2016 might have changed syphilis patients' healthcare seeking patterns, away from STI clinics to hospital-based facilities where penicillin was still available. The RésIST sentinel network, mainly based on STI clinics, could not grasp these shifting healthcare seeking patterns for syphilis.

Among MSM, the increase observed between 2014 and 2016 concerned not only gonorrhoea (a twofold increase in the number of cases), but also LGV. Despite the heterogeneity of observation systems, our results are in line with European data and overall trends [9-12]. Nevertheless, while the spread of STI was also considerable in MSM in the United Kingdom (UK), with an increase of 105% in gonorrhoea cases, of 151% in LGV cases and of 95% in syphilis cases between 2012 and 2015, the 2016 data showed a 22% decrease in gonorrhoea diagnoses, a 2.5% decline in LGV diagnoses but a 14% increase in syphilis cases [12-16]. Further data are needed to assess whether these decreases are due to improvements in testing, access to PrEP or a declining transmission.

The elevated levels of HIV co-infection in MSM diagnosed with rectal LGV, syphilis or gonorrhoea reflect insufficient condom use by HIV-positive MSM, a situation which has been observed in behavioural studies for many years [9-11]. The existence of rapid chains of STI transmission via sexual networks comprising HIV-positive MSM might contribute to the particular situation observed in this population [10]. This underlines the importance of ensuring that partners are tested, and the necessity of investigating spatio-temporal aggregates in order to interrupt STI transmission. Moreover, there is a need to assess the effect that other behavioural dimensions such as the use of geospatial networking applications, sero-adaptive behaviours and chemsex have on STI epidemics [17,18]. As these issues are insufficiently covered by current surveillance data in France, the section of the RésIST questionnaire dealing with sex venues was expanded in 2016 to take into account geospatial networking applications. Their effect on STI trends will be interpreted in the coming years. Concerning recreative drug use and especially chemsex, repeated behavioural surveys among patients are being considered to analyse the impact of chemsex on STI epidemics.

In heterosexual women and men, an increase in the number of gonorrhoea observed since 2012, was confirmed between 2014 and 2016, despite overall small numbers. Considering that chlamydial infections are the only STI monitored that predominantly occurs in women, it is possible to assume that the level of chlamydia transmission in heterosexual populations remains uncontrolled and high in France. Moreover, these STI affected the population aged 15–24 years in particular, hence the importance of a consistent testing strategy in the 15–24-year-olds to prevent potential consequences on fertility. Nevertheless, a systematic testing strategy was primarily applied to asymptomatic young women in STI clinics and family planning centres until 2018, in contrast to private medicine in France where a smaller proportion of asymptomatic cases was diagnosed [1-3]. The percentage of asymptomatic infections in women also decreased over the period 2014 to 2016, reflecting in part a decreasing compliance with this testing strategy.

The high representation of women among reported cases in France reflects the situation in Europe where most of declared cases come from heterosexual intercourse [9-12]. At the European level, the number of cases of chlamydia has remained stable since 2009 [9,10]. The characteristics of these patients (predominantly women aged 15–24 years) reflect testing recommendations and current practices in the reporting countries [9,10,15,21]. In the UK, a decrease in the number of cases observed since 2014 has been attributed to a reduction in testing coverage and changes in the way chlamydia data are reported and analysed [9,14,15].

As systematic chlamydia testing does not target MSM in France, and the national health insurance does not systematically reimburse the processing of three types of specimen (pharyngeal, uro-genital and anal) per patient, an underrepresentation of the spread of chlamydial infections in this population – in particular infections at extra-genital locations – cannot be dismissed [3]. If testing recommendations are applied, they certainly impact STI epidemiology by intensifying testing and screening of asymptomatic individuals, although their sustainability in the long term and their suitability considering current epidemiology and improvements in diagnostic tests (e.g nucleic acid amplification test (NAAT)) might be a concern [3-8]. Nevertheless, these recommendations remain insufficiently known outside STI clinics and specialised health facilities. A global approach to STI testing including systematic sampling from multiple anatomical locations and initiatives encouraging self-sampling and community-based testing, could contribute considerably to the control of STI. A population-based revision of the national recommendations, aiming to increase the yearly volume of systematic STI testing in exposed populations or in case of unprotected sex, might help to control STI epidemics.

Reimbursement of chlamydial NAAT by the national health insurance since 2010 and the spread of NAAT combining chlamydia and gonorrhoea testing probably played a role in the increase in asymptomatic cases. NAAT also made anatomical multisite testing easier thanks to its greater sensitivity; however, urogenital specimens therefore remain predominant [1]. Moreover, while NAAT for chlamydial infections has been reimbursed since 2010, patients had to cover the whole cost of NAAT prescribed for gonorrhoea until 2018. However, the amount of diagnoses in asymptomatic people remains insufficient to control the ongoing epidemics. PrEP will also provide an opportunity for more frequent STI testing, mostly in MSM, which could lead to an initial rise in the number of STI diagnoses followed by a decrease as more patients are treated and transmission chains are broken [19,20]. Modelling might help define precise testing goals.

Regarding gonorrhoea, France has seen a moderate increase in sensitivity to third-generation cephalosporins (cefixime or ceftriaxone). The average minimum inhibitory concentrations of cefixime and ceftriaxone continue to decrease. Two strains resistant to ceftriaxone were isolated in 2010 and another one in 2017 [1,22,23]. A decrease in cefixime and ceftriaxone resistance is also observed in European countries where azithromycin and ciprofloxacin resistance remains high [24]. Even though our results confirm a favourable trend observed for many years, surveillance of gonorrhoea antibiotic sensitivity is essential to prevent the spread of multi-resistant strains given its increasing transmission, notably in MSM [2,22-26]. Healthcare providers should be particularly vigilant and continue to prescribe cultures and sensitivity testing, in the context of the rising importance of molecular diagnostic techniques.

## Conclusion

STI diagnoses continue to increase in France, particularly in MSM, and this may be related to an increase in risky sexual behaviours in this population. Regular and early testing of patients and their partners, followed by rapid treatment, is therefore essential to interrupt STI transmission. With the advent of PrEP, epidemiological surveillance needs to be adapted to accurately measure its potential effects on the dynamics of STI. Reinforcing STI surveillance and investigating spatiotemporal clusters could also provide a better understanding of how to prevent STI at a subnational level. Systematic data collection from STI clinics, and sentinel surveillance networks, use of healthcare reimbursement data and implementation of specific surveys could together contribute to robust national and subnational indicators in a context of elevated incidences, where paper-based forms become less relevant to monitor STI trends.

## Referents for the regional offices of the French national public health agency

Lyderic Aubert, Emmanuel Belchior, Aurélie Etienne, Bertrand Gagnière, Anne Guinard, Mathilde Melin, Bakhao N’Diaye, Nathalie Nicolay, Ronan Ollivier, Frederic Pages, Laurence Pascal, Marion Petit Sinturel, Marie-Eve Raguenaud, Cyril Rousseau, Jeanine Stoll, Sabrina Tessier, Alexandra Thabuis, Jenifer Yai.
